# Inhibitory attentional control in anxiety: Manipulating cognitive load in an antisaccade task

**DOI:** 10.1371/journal.pone.0205720

**Published:** 2018-10-16

**Authors:** Julian Basanovic, Lies Notebaert, Patrick J. F. Clarke, Colin MacLeod, Philippe Jawinski, Nigel T. M. Chen

**Affiliations:** 1 Centre for the Advancement of Research on Emotion, School of Psychological Science, The University of Western Australia, Crawley, Australia; 2 School of Psychology, Curtin University, Bentley, Australia; 3 Department of Psychology, Humboldt-Universität zu Berlin, Berlin, Germany; 4 School of Occupational Therapy, Social Work and Speech Pathology, Curtin University, Bentley, Australia; University of Verona, ITALY

## Abstract

Theorists have proposed that heightened anxiety vulnerability is characterised by reduced attentional control performance and have made the prediction in turn that elevating cognitive load will adversely impact attentional control performance for high anxious individuals to a greater degree than low anxious individuals. Critically however, existing attempts to test this prediction have been limited in their methodology and have presented inconsistent findings. Using a methodology capable of overcoming the limitations of previous research, the present study sought to investigate the effect of manipulating cognitive load on inhibitory attentional control performance of high anxious and low anxious individuals. High and low trait anxious participants completed an antisaccade task, requiring the execution of prosaccades towards, or antisaccades away from, emotionally toned stimuli while eye movements were recorded. Participants completed the antisaccade task under conditions that concurrently imposed a lesser cognitive load, or greater cognitive load. Analysis of participants’ saccade latencies revealed high trait anxious participants demonstrated generally poorer inhibitory attentional control performance as compared to low trait anxious participants. Furthermore, conditions imposing greater cognitive load, as compared to lesser cognitive load, resulted in enhanced inhibitory attentional control performance across participants generally. Crucially however, analyses did not reveal an effect of cognitive load condition on anxiety-linked differences in inhibitory attentional control performance, indicating that elevating cognitive load did not adversely impact attentional control performance for high anxious individuals to a greater degree than low anxious individuals. Hence, the present findings are inconsistent with predictions made by some theorists and are in contrast to the findings of earlier investigations. These findings further highlight the need for research into the relationship between anxiety, attentional control, and cognitive load.

## Introduction

An essential function of effective attentional processing is the capacity to control the allocation of attention in the presence of task-irrelevant information. This function, known as attentional control, involves several components of cognitive processing including the attentional inhibition of distractor stimuli, volitional control of the allocation of attention towards target stimuli, and working memory necessary for the active maintenance of attentional processing priorities [1]. Importantly, individuals differ in the degree to which they are effective at implementing attentional control. One factor that has been demonstrated to affect attentional control performance is individual differences in the tendency to experiencing anxiety (anxiety vulnerability).

Researchers have revealed that, in general, heightened anxiety vulnerability is associated with poorer attentional control, and specifically a reduction in the capacity to control the attentional inhibition of task-irrelevant stimuli (see [2] for a review). Evidence of this effect has commonly been observed using an antisaccade task paradigm [3]. The antisaccade task paradigm requires participants to execute eye movements (saccades) either towards or away from an abruptly presented stimulus. The movement to attend towards the stimulus (i.e. a prosaccade) is largely a reflexive response and provides an assessment of stimulus driven attentional capture. By contrast, the correct execution a saccade away from the stimulus (i.e. an antisaccade) requires control over the inhibition of the reflexive prosaccade and the execution of a volitional saccade away from the stimulus. The difference in the latency to execute antisaccades relative to prosaccades, the ‘antisaccade cost’, has commonly been taken to reflect the ability of individuals to exert inhibitory attentional control, with larger costs indicating poorer capacity to exert control [4–6].

To examine anxiety-linked differences in inhibitory attentional control, Derakshan and colleagues [7] had individuals who varied in anxiety vulnerability complete an antisaccade task. The investigators found that individuals high in anxiety vulnerability as compared to those low in anxiety vulnerability demonstrated a significantly greater antisaccade cost, indicative of an anxiety-linked reduction in the capacity to control attentional inhibition. Subsequent studies have demonstrated evidence of anxiety-linked impairments in inhibitory attentional control across variants of the antisaccade task paradigm [8,9], and within other attentional tasks. For example, Moser et al. examined the association between level of anxiety vulnerability and the degree to which task-irrelevant distractor stimuli captured attention during a visual search task [10]. It was found that heightened anxiety vulnerability was associated with elevated attentional distraction by the distractor stimuli. Similarly, Edwards et al. showed that heightened anxiety vulnerability was associated with reduced inhibitory control during a No-Go paradigm, which requires participants to execute volitional responses on target-absent trials and inhibit responding on relatively rare and randomly presented target-present trials [11]. Given these and other findings demonstrating association between elevated anxiety vulnerability and reduced inhibitory attentional control performance (e.g. [9,12,13]) scholars have theorised that heightened anxiety vulnerability impairs inhibitory attentional control performance [2,14,15].

While researchers have demonstrated that increasing load on perceptual processing can increase performance on primary cognitive tasks under certain conditions [16,17], it is also well documented that performance on a primary cognitive task can be adversely impacted as demands on cognitive processes, often manipulated with a concurrent secondary task, are increased [18]. For example, investigators have observed that antisaccade performance decreases when individuals are required to perform a secondary task that imposes high demands on working memory, as compared to low demands on working memory, reflecting an adverse impact of elevated cognitive load upon inhibitory attentional control [19–21]. Such findings have led theorists to propose that individual differences in inhibitory attentional control performance result from the degree to which cognitive capacity is able to be recruited [22]. Accordingly, theorists interested in the relationship between anxiety vulnerability and attentional control performance have hypothesised that anxiety-linked reduction in attentional control performance may arise due to anxiety-linked reductions in available cognitive capacity [2,23]. This has given rise to the specific prediction that the adverse impact of a concurrent cognitive load upon inhibitory attentional control performance should be greater for individuals with heightened anxiety vulnerability, as compared to individuals with low anxiety vulnerability [23].

Researchers have sought to test the validity of this prediction by examining the impact of increasing cognitive load on anxiety-linked impairment in inhibitory attentional control. These investigations have resulted in mixed findings. Berggren et al. required participants to complete an antisaccade task while engaging in a secondary task requiring the detection of auditory tones (low cognitive load), or identification of the tone’s pitch (high cognitive load) [24]. In this case, increased cognitive load resulted in reduced task performance across all participants. Importantly however, when considering participants’ anxiety, it was revealed that greater levels of anxiety vulnerability were associated with greater declines in attentional control performance during the high cognitive load condition, relative to the low load condition. This finding is consistent with the prediction that elevation in cognitive load would disproportionately deteriorate inhibitory attentional control performance amongst high anxious individuals, as compared to low anxious individuals. Critically however performance on the secondary task was not measured. Thus, it is unclear whether these findings were the result of the predicted effect, or the result of differences in the degree to which individual with higher or lower levels of anxiety vulnerability engaged with the secondary task.

Another investigation of this prediction was conducted by Berggren et al. [25]. Here, participants were required to engage in a visual search task in which participants were instructed to determine whether one face, in a display of eight faces, displayed a unique emotional expression. A within-participants manipulation of cognitive load either required participants to concurrently engage in a counting task that required counting backwards from a given number in intervals of three (high cognitive load) or did not require engagement with a concurrent task (low cognitive load). Results revealed that high anxious individuals demonstrated reduced visual search performance, reflected by longer search latencies, under conditions of high cognitive load as compared to low cognitive load, whilst low anxious individuals did not show this effect. This finding too is consistent with the prediction that elevated cognitive load will impact inhibitory attentional control performance disproportionately for individuals high in anxiety vulnerability as compared to low in anxiety vulnerability. That is, if indeed high anxious participants, as compared to low anxious participants, were impaired at inhibiting the processing of faces in the set, this would foreseeably lead to relatively longer search latencies. Importantly however, numerous attentional processes are believed to be involved in the efficiency and speed of visual search during visual search paradigms, including attentional capture and attentional engagement [26], attentional disengagement [27], and attentional inhibition [28]. Thus, the results reported by Berggren et al. [25] could also be consistent with alternative explanations. For example, it is possible that anxiety-linked differences observed in visual search latencies were the result of differences in the speed at which participants disengaged attention from faces in the set once they had attended to them, rather than differences in inhibiting attentional capture by faces in the set. Once again, while this finding is consistent with the proposal that elevated cognitive load disproportionately impacts inhibitory attentional control amongst high anxious individuals, the possibility that observed visual search performance was the result of cognitive load impacting other attentional processes cannot be ruled out.

Nonetheless, the findings by Berggren et al. [24,25] are in contrast with other studies, which have not observed an adverse effect of heightened cognitive load on inhibitory attentional control performance amongst high state-anxious individuals. Najmi et al. [29] examined performance on the Attention Network Task [30], a task designed to assess specific facets of attentional processing, including inhibitory attentional control. Cognitive load was manipulated by modifying the difficulty of a reverse counting task designed to engage working memory resources. Importantly, investigators observed no significant difference in inhibitory attentional control performance between conditions of low and high cognitive load for low state-anxious individuals, while observing that high state-anxious individuals demonstrated increased inhibitory attention control performance under high cognitive load, as compared to low cognitive load. It is important to note however that this study examined individuals who rated high or low in state anxiety, not anxiety vulnerability (although such measures are known to be highly correlated). As such, these findings do not conclusively inform upon the relationship between cognitive load and inhibitory attentional control performance amongst individuals who are high in anxiety vulnerability. Furthermore, the study did not measure participants’ adherence to the reverse counting task, meaning that individual differences in inhibitory attentional control performance under each cognitive load condition could potentially be the result of differences in adherence to the cognitive load task under each condition.

It is also noteworthy that there is evidence that the emotional tone of attentional stimuli can modulate the magnitude of anxiety-linked impairment in inhibitory attentional control [7,12,31–33], and so it is plausible that conditions that load cognitive resources may further exacerbate the impact of emotional stimuli on attentional control performance. Indeed, some researchers have theorised that as attentional control become more greatly impaired emotionally negative stimuli will capture attention to a greater degree [34], while other have proposed that, when cognitive processes are taxed emotional stimuli will become less salient to the attentional system [35,36]. Given these alternate predictions, investigation of the impact of emotionally negative stimuli on anxiety-linked differences in attentional control performance in the face of high and low cognitive load would usefully inform theories that describe the manner in which elevated anxiety impacts controlled attentional processing of emotional information [2,34].

Investigation of the effect of cognitive load on the relationship between anxiety vulnerability and attentional control performance is critical to understanding the mechanisms underlying anxiety-linked impairments in attentional control performance, as well as to further informing theories that seek to describe the relationship between attentional control and anxiety vulnerability (e.g. [15,23]). Thus, given the methodological issues present in previous studies and the inconsistency in the resulting findings it is important that research continues to investigate the relationship between inhibitory attentional control, cognitive load, and anxiety vulnerability.

Hence, the aim of the present study was to contribute to the investigation of this relationship by employing a methodology that could overcome the limitations of previous research. To do so, the present study subjected individuals with high levels and low levels of anxiety vulnerability to a dual task paradigm that could assess inhibitory attentional control whilst concurrently manipulating cognitive load. To overcome the limitations of previous research it was necessary for this methodology to implement a task capable of assessing inhibitory attentional control specifically. Hence, inhibitory attentional control was assessed using an antisaccade task, which requires participants to execute eye movements either towards or away from an abruptly presented stimulus. As described earlier, this task has been commonly used to assess inhibitory attentional control and the impact of anxiety vulnerability on this attentional process [7–9,37].

To assess the impact of cognitive load on attentional control, participants’ performance on the antisaccade task was assessed under conditions of high cognitive load and low cognitive load. Once more, to overcome the limitations of previous research it was essential that participant performance to this secondary task could be examined. Thus, the methodology manipulated cognitive load by requiring participants to retain a set of digits in memory for later recall. Cognitive load was manipulated by increasing or decreasing the number of unique digits to be retained in memory in each set and performance was examined by assessing the rate of accurate recall.

Lastly, given evidence that anxiety-linked reduction in inhibitory attentional control may be modulated by the emotional tone of attentional stimuli [7,31–33], and given that theorists have sought to describe the manner in which elevated anxiety vulnerability impacts controlled attentional processing of emotional information [2,34,38], it was deemed useful to permit examination of the effect of cognitive load on anxiety-linked differences in attentional control performance in the face of emotional and unemotional stimuli. Hence, the emotional tone of stimuli presented in the antisaccade task was varied within participants to present either emotionally negative, emotionally positive, or unemotional attentional stimuli.

Considering previous research that has demonstrated anxiety-linked impairment in inhibitory attentional control, it was predicted that high anxious individuals, when compared to low anxious individuals, would exhibit reduced inhibitory attentional control performance, as indexed by reduced performance on the antisaccade task. Because engagement with a concurrent working memory task will occupy cognitive resources amongst all participants, it was also predicted that conditions imposing a heightened cognitive load, as compared to a lesser cognitive load, would result in reduced inhibitory attentional control performance across individuals irrespective of level of anxiety vulnerability. Crucially however, the purpose of the present study was to determine the validity of the prediction that the adverse impact of increasing cognitive load upon attentional control performance would be greater for individuals with heightened anxiety vulnerability, as compared to individuals with low anxiety vulnerability. Hence, the primary concern of the study was to determine whether high anxious individuals, when compared to low anxious individuals, demonstrate disproportionately reduced attentional control performance under conditions that impose a high cognitive load, relative to those imposing low cognitive load.

## Method

### Participants

In order to selectively recruit individuals with relatively high and low levels of anxiety vulnerability, an initial screening procedure was conducted on 644 undergraduate students using the Trait scale of the State Trait Anxiety Inventory (STAI-T; [39]). This procedure was conducted as part of a larger research screening battery completed by undergraduate psychology students at the University of Western Australia. The STAI-T is a widely used assessment for the measurement of anxiety vulnerability. Individuals with STAI-T scores in the upper and lower tercile, corresponding to scores above 43 and below 36 respectively, were then invited to participate in the present study in exchange for course credit or AUD$10. Twenty-five high trait anxious (18 female) and 24 low trait anxious (14 female) participants were recruited to complete the study. All participants had normal vision or corrected-to-normal vision.

### Experimental hardware

The antisaccade task was developed using Experiment Builder 1.10.165 (SR Research Ltd, Mississauga, Canada) and was presented on a widescreen 24” monitor at a resolution of 1920 x 1080 pixels. Participants’ eye movements were recorded using a desk-mounted SR Research EyeLink 1000. This eye tracker recorded monocular gaze at 1000Hz, with up to .25° accuracy and .01° spatial resolution. Eye movements were recorded using pupil centre corneal reflection with nine calibration points.

### Experimental stimuli

#### Antisaccade task stimuli

Antisaccade task stimuli consist of 16 actors, eight male and eight female, each expressing happy and angry emotions, as well as a neutral pose. Face images were taken from the NimStim Set of Facial Expressions [40]. Images in the set have demonstrated a high level of reliability and validity in the recognition of these facial expressions by observers [40]. Face images were presented in greyscale and were 5.47cm x 7.03cm in size, subtending at approximately 5.80° x 7.46° visual angle, at a viewing distance of 54cm. In order to control for the potential extraneous influence of low-level image properties on saccadic behaviour, face images were matched on mean luminance and contrast using the SHINE toolbox for MATLAB [41].

#### Cognitive load stimuli

Cognitive load stimuli for the antisaccade task were employed to impose either high or low cognitive load. These stimuli consisted of sets of 6 randomly generated digits presented in a row. Each digit in the set was presented in white text, and ranged from 0 to 9. The row of digits spanned 4.81cm x .92cm in size, which subtended a visual angle of approximately 5.10° by .98° respectively. Under conditions of high cognitive load, each digit set consisted of six unique digits (e.g. 859024). Under conditions of low cognitive load, the digit set consisted of a single digit repeated six times (e.g. 333333). This method of presentation manipulated cognitive load while keeping perceptual load constant across conditions.

### Antisaccade task

The purpose of the antisaccade task was to yield a measure of participants’ inhibitory attentional control performance under conditions that imposed a concurrent lower or heightened cognitive load. The task required participants to execute saccades towards (prosaccades), or saccades away from (antisaccades), a face stimulus.

For each trial, a central fixation cross was initially presented for 500ms. The fixation cross was white and subtended at approximately 1° VA. This was replaced by a high load or low load cognitive load stimulus for 2000ms, followed by a mask (“######”) for 200ms. A second fixation cross was then presented for 1500ms, followed by the presentation of a face stimulus. A gaze contingency algorithm was implemented such that the face stimulus would only appear if a fixation was detected at the location of the cross, otherwise the cross remained on the screen until an appropriate fixation was detected. The face stimulus was displayed for 600ms at 11° VA eccentricity to the left or right of the screen centre with equal frequency. The stimulus displayed a happy, neutral, or angry face with equal frequency. During presentation of the face stimulus, participants were required to perform either a prosaccade or antisaccade in response to the stimulus. Following this, a probe was presented at the screen centre. The probe consisted of a single digit that was either present or not present in the preceding cognitive load stimulus with equal frequency. Participants were required to determine whether the probe was present. On trials where the probe was present in the cognitive load stimulus, the probe was present at any of the six positions within the cognitive load stimulus with equal probability. The probe was displayed until participants made their response with a corresponding button press. Subsequently the next trial was initiated following a 500ms inter-trial interval.

Eight blocks of trials were presented in a random order. The trial blocks orthogonally contrasted the saccade response and cognitive load required during trials. This resulted in two blocks requiring prosaccades under low cognitive load, two blocks requiring prosaccades under high cognitive load, two blocks requiring antisaccades under low cognitive load, and two blocks requiring antisaccade under high cognitive load. Each block contained 24 randomized trials. At the start of each block, the instruction “TOWARDS” or “AWAY” was presented to indicate the saccade response required to be executed in retaliation to the stimuli for each trial within the block.

On each trial the latency for participants to execute the required saccade was recorded. The latency to execute a saccade was defined as the interval between target onset and the initiation of the required saccade on each trial. Erroneous saccades and errors in probe responses were also recorded.

### Procedure

All procedures conducted were approved by the Human Research Ethics Committee at the University of Western Australia. Prior to recruitment, participants were provided an information sheet that informed them of the requirements involved in participation and briefed that the study sought to examine the manner in which the visual information processing system responds to images of faces under a variety of conditions. Participants were not made aware of the method or motive of participant recruitment and selection or the role of trait anxiety in the experimental design. Upon recruitment, participants provided written consent and basic demographic information. Next, participants were seated in front of the eye tracker with their head secured in a chin rest, at an approximate viewing distance of 54cm, and an eye-movement calibration procedure was conducted. The testing session was conducted in a sound attenuated room, with the experimenter monitoring participants’ eye movements from a second computer. Participants were verbally instructed of the requirements of the antisaccade task. Once participant understanding of the task was confirmed by the experimenter participants completed 16 practice trials. During these practice trials, the execution of each saccade and probe response was followed by the word “CORRECT” or “INCORRECT” for 1000ms in green or red colour respectively. This provided feedback to further ensure comprehension of the task requirements. After completing the practice trials, participants then completed the antisaccade task with no feedback provided. Re-calibration of eye-movement measures was performed throughout the task as needed. Once the experimental session had concluded the experimenter verbally debriefed participants as to the specific aim of the experiment and participants were provided this information in writing.

### Data preparation

Four participants failed to register a recordable gaze signal during the initial calibration procedure. An additional two participants did not comply with task instructions, each demonstrating a near 100% erroneous saccade rate across blocks in the antisaccade task. Analyses were conducted on the remaining 43 participants (23 high anxious, 16 female; 20 low anxious, 13 female). Analyses confirmed that remaining high anxious and low anxious participant groups did not significantly differ in age (M = 18.81, SD = 2.85 years), *t*(41) = 1.73, p = .092, or gender ratio, *χ*^2^(1, N = 43) = .10, p = .50, and continued to differ in level of trait anxiety (Low Anxiety Group, M = 33.75, SD = 6.03; High Anxiety Group, M = 46.57, SD = 8.19), *t*(41) = 5.77, p < .001, as intended.

Raw gaze samples were initially cleaned using a two-sample noise reduction filter [42]. Saccades were then defined as samples exceeding a 30°s^-1^ velocity threshold and 8000°s^-2^ acceleration threshold. To remove artefactual gaze data, such as anticipatory saccades, analysis of saccade measures included only those trials where the first saccade following target onset was greater than 3° in amplitude, had occurred between 83ms and 600ms following target onset, and was directed within 45° from horizontal. In addition, to ensure that saccade data reflected instances where participants had engaged in processing of the cognitive load stimulus, only trials with correct responses to the probe were included in analyses of saccade measures.

Of primary interest to the analysis was the measure of inhibitory attentional control. To derive such a measure, antisaccade cost scores was computed in keeping with a procedure adopted by other researchers who examined individual differences in antisaccade performance [6,31,43,44]. These antisaccade cost scores represented the difference, in milliseconds, between the mean antisaccade latency and the mean prosaccade latency within each task condition. Hence, a higher antisaccade cost score reflected relatively greater eye movement latencies associated with performing antisaccades as compared to prosaccades, and therefore reflects relatively poorer inhibitory attentional control performance.

## Results

It was important to ensure that any anxiety-linked differences in performance on the antisaccade task was not confounded by anxiety-linked differences in performance in the concurrent cognitive load task. Hence, analyses of the collated data first examined whether anxiety groups differed in their accuracy to discriminate the presence or absence of probes in the digit sets. Of primary importance to the present predictions, analyses next examined anxiety-linked differences in performance on the antisaccade task, as indexed by computed antisaccade costs scores, under each cognitive load condition and under conditions where face stimuli varied in emotional tone. Lastly, while saccade latencies represent the key measure of performance reflecting inhibitory attentional control during the antisaccade task, some investigators have reported anxiety-linked differences in the number of erroneous saccades performed during antisaccade tasks [12,33], and thus, analyses also examined the potential influence of emotional tone, cognitive load, and anxiety vulnerability on the number of erroneous saccades performed by participants during the antisaccade task. Analyses were conducted in *R*, using the *ez*, *lme4*, and *car* packages.

### Memory probe error rates

**Fig 1:**
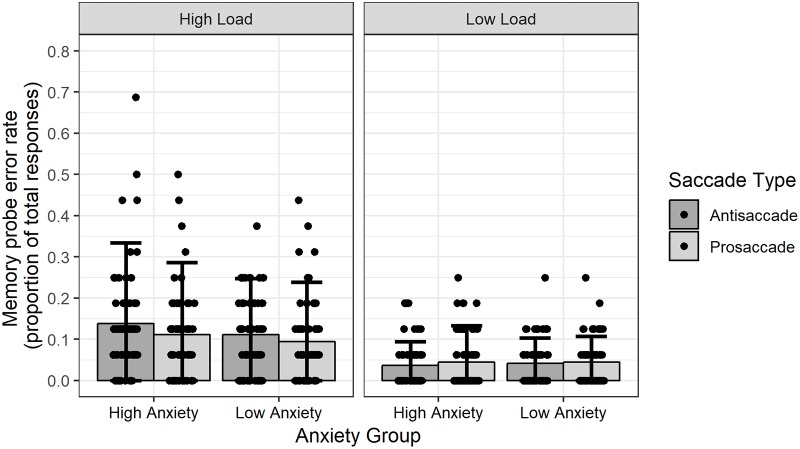
Memory probe error rates, as a proportion of total trials, for task conditions and anxiety groups. Height of bars represent mean error rates, error bars represent 95% confidence interval, points represent mean error rates of individual participants.

Across all conditions the average memory probe error rate was 7.80% (*SD* = 9.14). The pattern of memory probe error rates is presented in Fig 1. As the error rate represent a binomial variable an analysis of variance was deemed inappropriate to examine differences between experimental conditions. Instead, a mixed-effects logistic regression model was computed. This model included participant memory probe error rates as the outcome variable, and Anxiety Group (high trait anxiety vs low trait anxiety), Saccade Type (prosaccade vs antisaccade), Cognitive Load (low load vs high load), and Stimulus Valence (positive vs neutral vs negative) as fixed-effect factors. Participants were included as a random-effect variable. The resulting model was examined via an analysis of deviance that utilised Wald tests to examine the effect of the fixed-effects factors on memory probe error rates. This analysis revealed a main effect of Cognitive Load, *χ*^2^(1, N = 43) = 135.02, *p* < .001. This effect was reflected in the observation that participants made a greater number of errors when the set to be remembered contained six unique digits in the high load condition (*M* = 11.41%, *SD* = 7.75), as compared to one unique digit in the low load condition (*M* = 4.20%, *SD* = 3.02). A main effect of Anxiety Group was not observed from the analysis, *χ*^2^(1, N = 43) = 0.73, *p* = .39. No other significant main effects or interaction effects were observed from this analysis. Therefore, the results of this analysis demonstrated that, as expected, digit sets in presented in the high cognitive load condition were more difficult to recall than digit sets in the low cognitive load condition, and furthermore, anxiety groups did not differ in their accuracy to determine whether probes were present in digit sets.

Though not related to the hypotheses at hand, it was considered that some readers may be interested in the influence of gender on memory probe error rates. As such, a post-hoc analysis that also incorporated participant gender (male, female) as a fixed-factor was conducted. This analysis resulted in a significant main-effect involving gender, *p* = .024, and an interaction effect involving anxiety group, cognitive load, and gender, *p* = .043. However, we advise appropriate caution in the interpretation of these effects due to the fact that condition sample sizes were reduced considerably in these analyses. We guide readers to the publicly available dataset if they wish to pursue this line of investigation further.

### Antisaccade cost scores

Analyses next examined anxiety-linked differences in performance on the antisaccade task. Descriptive statistics of participants’ saccade latencies for each task condition and anxiety group are provided in Table 1.

**Table 1:** Descriptive statistics of participants’ mean saccade latency, in milliseconds, for each task condition and anxiety group.

Cognitive Load	Stimulus Valence	Saccade Type	Low Anxiety Group M (SD)	High Anxiety Group M (SD)
Low	Angry	Prosaccade	174.25 (32.92)	166.00 (37.13)
		Antisaccade	259.15 (28.57)	278.83 (47.63)
	Neutral	Prosaccade	168.72 (26.81)	160.10 (23.68)
		Antisaccade	250.89 (33.86)	263.00 (43.59)
	Happy	Prosaccade	169.31 (25.38)	167.26 (31.88)
		Antisaccade	246.28 (22.56)	274.46 (44.73)
High	Angry	Prosaccade	173.02 (32.73)	160.07 (28.73)
		Antisaccade	244.35 (31.50)	256.92 (42.41)
	Neutral	Prosaccade	170.79 (33.32)	156.69 (27.72)
		Antisaccade	245.34 (30.38)	260.20 (42.15)
	Happy	Prosaccade	175.35 (26.69)	160.63 (31.01)
		Antisaccade	243.24 (36.45)	264.49 (38.97)

**Fig 2:**
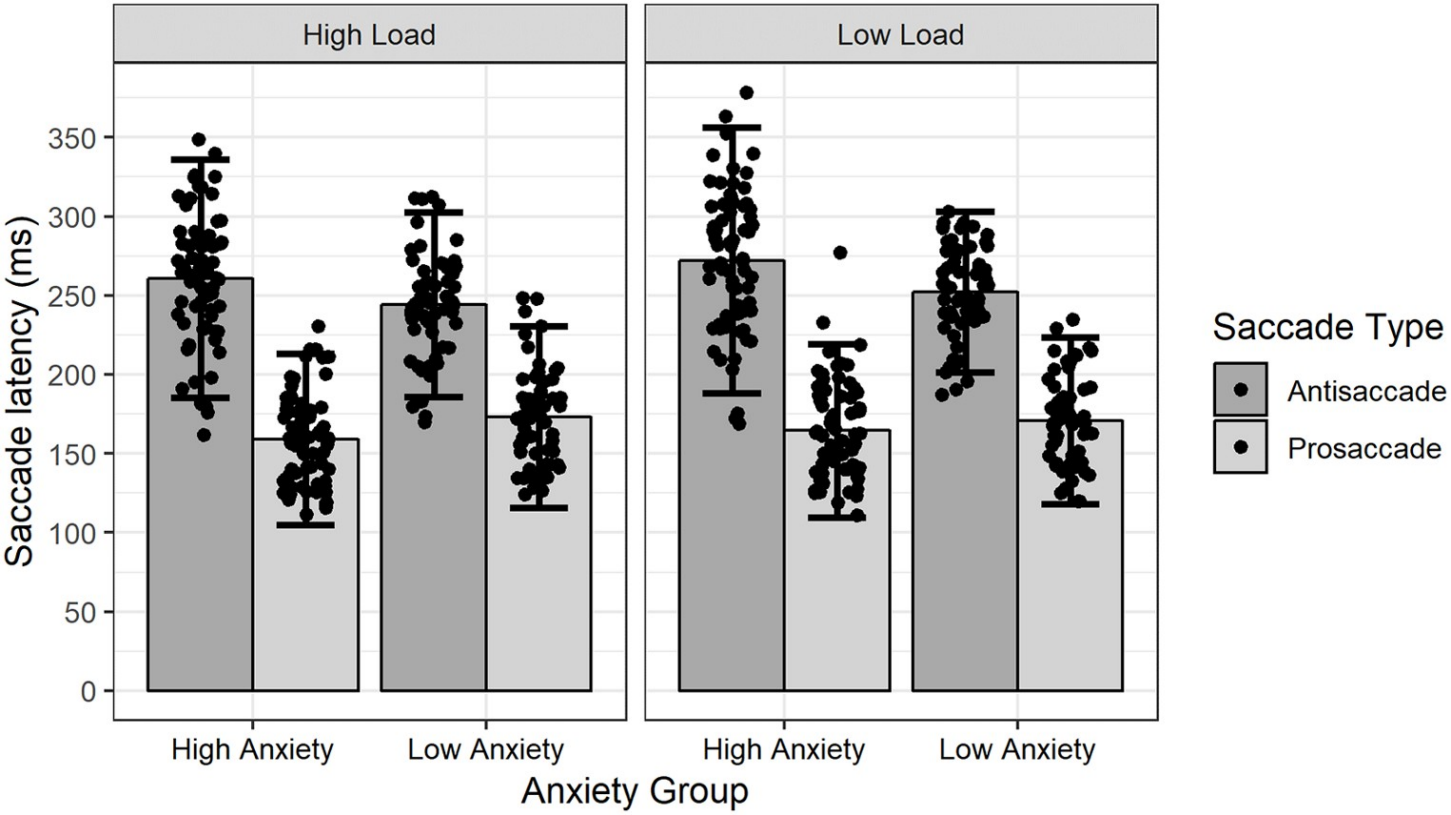
Saccade latencies, in milliseconds, for task conditions and anxiety groups. Height of bars represent mean latencies, error bars represent 95% confidence interval, points represent mean latencies of individual participants.

To examine the influence of anxiety vulnerability and cognitive load on antisaccade performance a mixed-design ANOVA was conducted on participants’ antisaccade costs scores, computed under each cognitive load condition and under conditions where face stimuli varied in emotional tone. This analysis included Anxiety Group (high trait anxiety vs low trait anxiety) as the between-groups factor, and Cognitive Load (low vs high) and Stimulus Valence (positive vs neutral vs negative) as between-groups factors. From the results of this analysis a main effect of Anxiety Group was evident, *F*(1,41) = 6.39, *p* = .015, η^2^ = .13. This effect indicated that, consistent with our predictions, high anxious individuals exhibited a greater antisaccade cost (*M* = 104.52, *SD* = 49.93) as compared to low trait anxious individuals (*M* = 76.30, *SD* = 53.54). The analysis also yielded a main effect of Cognitive Load, *F*(1,41) = 7.29, *p* = .010, η^2^ = .15. This effect revealed that, in contrast to our predictions, high cognitive load resulted in a smaller antisaccade cost (*M* = 87.38, *SD* = 41.82) as compared to low cognitive load (*M* = 95.41, *SD* = 38.12) for participants generally. Most importantly however, the analysis did not yield a significant interaction effect involving Anxiety Group and Cognitive Load, *F*(1,41) = 0.40, *p* = .53, η^2^ = .01. No other effects reached statistical significance. Crucially, this revealed that the effect of increased cognitive load on antisaccade performance did not significantly differ amongst high and low anxious participants. No other significant effects emerged from this analysis. The pattern of data that gave rise to these effects is presented in Fig 2.

Once more, though not related to the hypotheses at hand it was considered that some readers may be interested in the influence of gender on antisaccade cost scores. As such, a post-hoc analysis that also incorporated participant gender (male, female) as a between groups factor was conducted. This analysis resulted in a significant interaction effect involving anxiety group, cognitive load, and gender *p* = .043. However, once again we advise caution in the interpretation of the results of this analysis as condition sample sizes were reduced considerably in these analyses, and we guide readers to the publicly available dataset if they wish to pursue this line of investigation further.

### Saccade error rates

**Fig 3:**
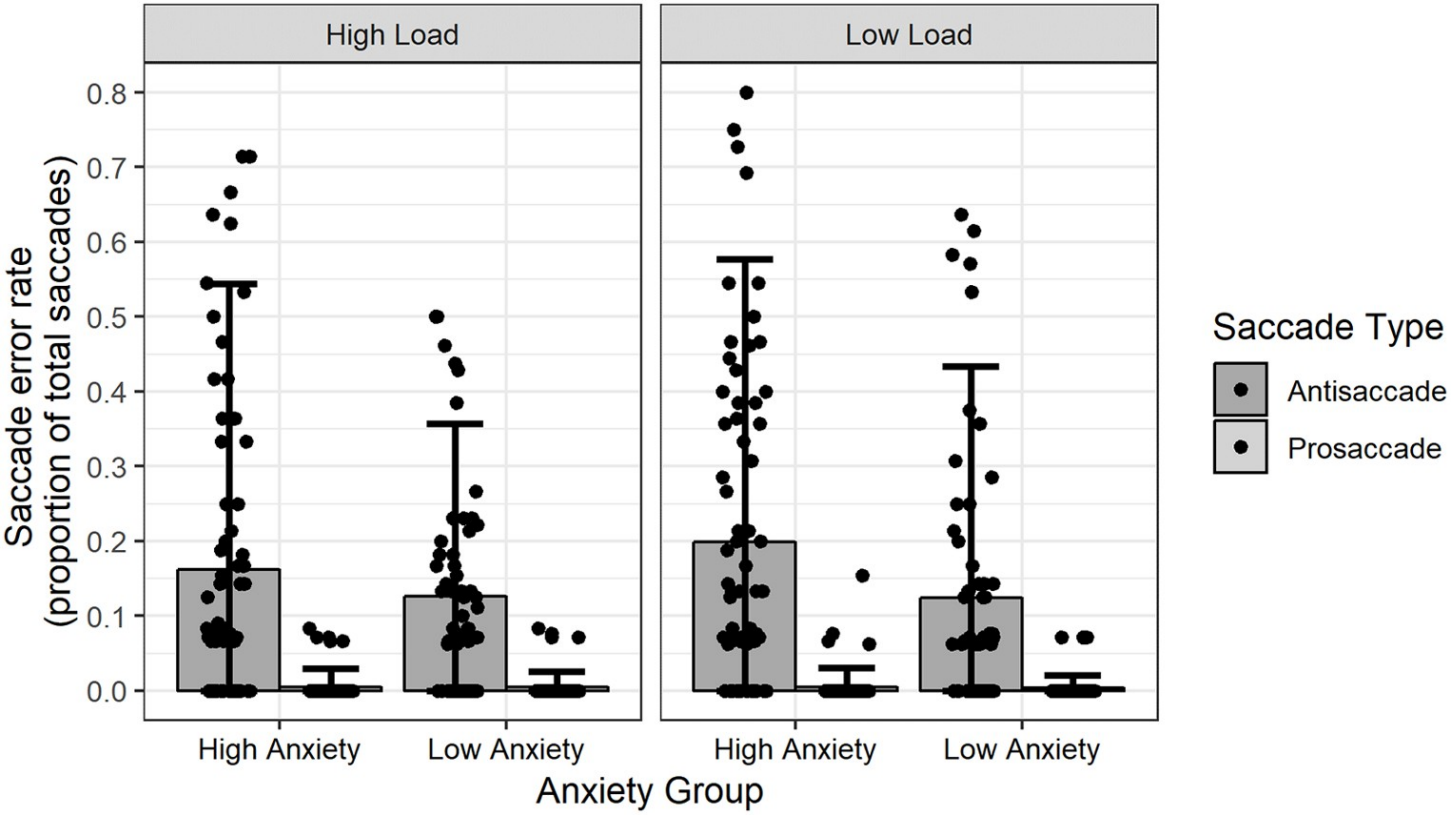
Saccade error rates, as a proportion of total trials, for task conditions and anxiety groups. Height of bars represent mean error rates, error bars represent 95% confidence interval, points represent mean error rates of individual participants.

Lastly, analyses examined the potential influence of emotional tone, cognitive load, anxiety vulnerability on the number of erroneous saccades performed by participants during the antisaccade task. The pattern of saccade error rates is presented in Fig 3. Across all conditions the average saccade error rate was 7.98% (*SD* = 15.27). Once again, as the error rate represents a binomial variable an analysis of variance was deemed inappropriate to examine differences between experimental conditions. Instead, a mixed-effects logistic regression model was computed. This model included participant saccade error rates as the outcome variable, and Anxiety Group (high trait anxiety vs low trait anxiety), Saccade Type (prosaccade vs antisaccade), Cognitive Load (low load vs high load), and Stimulus Valence (positive vs neutral vs negative) as fixed-effect factors. Participants were included as a random-effect variable. The resulting model was examined via an analysis of deviance that utilised Wald tests to examine the effect of the fixed-effects on saccade error rates. Only a main effect of Saccade Type was evident *χ*^2^(5, N = 43) = 200.07, *p* < .001. This effect was reflected by the observation that, in general, participants made a greater number of erroneous saccades on trials that required the execution of an antisaccade (*M* = 15.48%, *SD* = 16.38) as compared to trials that required the execution of a prosaccade (*M* = .48%, *SD* = .85). A main effect of Anxiety Group was not observed from the analysis, *χ*^2^(3, N = 43) = 1.40, *p* = .70, nor any other significant main effects or interaction effects. Thus, this analysis revealed that neither cognitive load condition or anxiety vulnerability impacted upon the number of erroneous saccades made by participants.

## Discussion

The present study examined the impact of increasing cognitive load on anxiety-linked differences in attentional control. As anticipated, the results revealed that high anxious participants demonstrated significantly greater antisaccade costs, as compared to low anxious participants, suggestive of poorer inhibitory attentional control. The study also found no effect of the emotional tone of attentional stimuli on anxiety-linked differences in inhibitory attentional control performance. Interestingly, and in contrast to previous research the findings also suggest that greater cognitive load may have facilitated attentional control performance. In general, participants were found to exhibit smaller antisaccade costs under conditions that imposed a higher cognitive load, relative to the low load condition. Of primary importance however, was the finding that the imposition of a higher cognitive load did not differentially impact inhibitory attentional control performance in high anxious and low anxious participants. Furthermore, the failure to observe such a difference could not be accounted for by anxiety-linked differences in engagement with cognitive load items or anxiety-linked differences in the number of erroneous saccades performed during the antisaccade task. These findings will now be discussed in turn.

The present observation that high anxious participants demonstrated significantly greater antisaccade costs as compared to low anxious participants is consistent with previous research on anxiety-linked differences in cognitive performance. Specifically, the present findings are consistent with earlier studies that have demonstrated association between elevated anxiety and reduced attentional control performance [7,9], as well as the proposal by theorists that individuals with heightened anxiety vulnerability, as compared to low in anxiety vulnerability, are characterised by reduced control of attentional inhibition [2,23].

In contrast, the present study did not replicate previous findings that have revealed anxiety-linked differences in attentional control performance to be modulated by the emotional tone of attentional stimuli [7,31,32]. However, while some studies have demonstrated that the presentation of stimuli containing negative emotional tone may result in further reduction in attentional control performance amongst high anxious individuals other studies have observed no such effect [33]. Further, the present findings support the proposal that emotional stimuli will become less salient to the attentional system when cognitive resources are taxed [35,36]. Researchers have also observed reduced processing of emotional information under conditions of heightened cognitive load. For example, Van Dillen et al. [45] demonstrated that increasing cognitive load resulted in down-regulation of neural responses associated with the processing of negatively valenced stimuli. Similarly, King and Schaefer [46] demonstrated that startle responses typically resulting from the viewing of negatively valenced images can be reduced under high cognitive load, indicative of reduced saliency of the emotional content of the images. Thus, it may be the case that in the present study the demands of the concurrent cognitive load task diminished the attentional salience of the emotional tone of the attentional stimuli.

Of primary importance however, the present findings differ from the hitherto inconsistent findings on the effect of increasing cognitive load on anxiety-linked reduction in attentional control performance. Berggren et al. [24,25] reported anxiety-linked impairments in attentional control to be disproportionately elevated when high and low anxious participants were subjected to a heightened cognitive load, relative to a low cognitive load. Conversely, Najmi et al. [29] reported that heightened cognitive load disproportionately improved the attentional control performance of participants with high anxiety vulnerability, as compared to low anxiety vulnerability. However, despite demonstrating anxiety-linked impairment in inhibitory attentional control performance, the present study demonstrated no effect of increasing cognitive load on the relative inhibitory attentional control performance of high anxious participants, as compared to low anxious participants. It is of note that the present methodology overcame limitations present in earlier studies. Specifically, the present study assessed individual differences in performance amongst individuals who differ in anxiety vulnerability rather than state-anxiety, was able to demonstrate that engagement in the cognitive load task was comparable between high and low anxious participants, and utilised an attentional task capable of specifically measuring individual differences in inhibitory attentional control amongst participants. Nonetheless, the inconsistency between the present findings and those of other researchers highlights a clear need for further examination into the effect of cognitive load on anxiety-linked differences in attentional control.

Importantly, the present findings also do not support predictions made by standing theories that propose anxiety-linked impairment in inhibitory attentional control is underpinned by anxiety-linked reductions in available cognitive capacity [15,23]. Specifically, these theories have made the prediction that under conditions of high cognitive demand tasks will more readily overload the cognitive capacity of high anxious individuals as compared to low anxious individuals, and that this impact of cognitive demand will results in even greater impairment in inhibitory attention control performance. It is possible however, the task used to impose a high cognitive load in the present study did not engage cognitive resources to a sufficiently high degree to overload the cognitive capacity of high anxious individuals. Thus, while high anxious participants demonstrated some impairment on inhibitory attentional control they were able to maintain attentional performance relative to low anxious participants when cognitive load was increased. Alternately, it is possible that the task used to manipulate cognitive load in the present task was indeed successful in doing so, though engaged a cognitive process that does not consume the same resources as those recruited in the performance of inhibitory attentional control.

Interestingly, the present finding that elevated cognitive load increased attentional control performance in the present study may help to identify the mechanisms that underpin anxiety-linked impairment in inhibitory attentional control. Other avenues of research investigating the role of cognitive load on attentional control have demonstrated that the imposition of a load that recruits processes to maintain visual representations of stimuli in working memory can result in improved performance on attentional tasks by reducing the attentional resources available to process distracting stimuli [16]. For example, across two studies Konstantinou et al. [47,48] assessed participants’ processing of attentional distractors that were presented during a response competition task. Participants concurrently adopted a cognitive load requiring either visual maintenance or verbal rehearsal processes. It was found that increasing load on visual maintenance led to greater inhibition of distractor stimuli, whereas increasing loads on verbal rehearsal processes did not. Thus, it is plausible that the method of imposing cognitive load in the present study predominantly consumed processes involved in visual maintenance and so attenuated the salience of the attentional stimuli that were present in the antisaccade task. Importantly, given that the present cognitive load manipulation failed to differentially impact attentional control amongst high and low anxious participants, this may indicate that anxiety-linked reduction in inhibitory attentional control performance is not underpinned by anxiety-linked reductions in cognitive processes associated with visual maintenance. Nonetheless, while this may serve as a potential explanation for the present findings there remains a clear need for researchers to continue to investigate the manner in which cognitive load impacts attentional control performance in high anxious individuals, so as to reveal the mechanisms that underpin anxiety-linked impairment in attentional control.

For the moment however, though the present study demonstrated evidence that heightened anxiety vulnerability is characterised by a decline in inhibitory attentional control performance, it did not demonstrate evidence that increasing cognitive load differentially impacted performance for individual’s high in anxiety vulnerability, as compared to low in anxiety vulnerability. While these findings support the proposal made by theorists that heightened anxiety vulnerability is characterised by reduced inhibitory attentional control, the findings sit contrary to the prediction that heightened cognitive load demands will disproportionately adversely impact inhibitory attentional control performance amongst individuals with heightened anxiety vulnerability, as compared to those low in anxiety vulnerability.
